# CpG location and methylation level are crucial factors for the early detection of oral squamous cell carcinoma in brushing samples using bisulfite sequencing of a 13-gene panel

**DOI:** 10.1186/s13148-017-0386-7

**Published:** 2017-08-15

**Authors:** Luca Morandi, Davide Gissi, Achille Tarsitano, Sofia Asioli, Andrea Gabusi, Claudio Marchetti, Lucio Montebugnoli, Maria Pia Foschini

**Affiliations:** 1 “M. Malpighi” Section of Anatomic Pathology, Department of Biomedical and Neuromotor Sciences, Bellaria Hospital, University of Bologna, via Altura n.3, 40137 Bologna, Italy; 2 Section of Oral Science, Department of Biomedical and Neuromuscular Sciences, University of Bologna, Bologna, Italy; 3 Unit of Maxillofacial Surgery, S. Orsola Hospital Bologna, Bologna, Italy

**Keywords:** Bisulfite sequencing, Quantitative DNA methylation analysis, Algorithm, Oral brushing, Oral squamous cell carcinoma

## Abstract

**Background:**

Oral squamous cell carcinoma (OSCC) is usually diagnosed at an advanced stage and is commonly preceded by oral premalignant lesions. The mortality rates have remained unchanged (50% within 5 years after diagnosis), and it is related to tobacco smoking and alcohol intake. Novel molecular markers for early diagnosis are urgently needed. The purpose of this study was to evaluate the diagnostic value of methylation level in a set of 18 genes by bisulfite next-generation sequencing.

**Methods:**

With minimally invasive oral brushing, 28 consecutive OSCC, one squamous cell carcinoma with sarcomatoid features, six high-grade squamous intraepithelial lesions (HGSIL), 30 normal contralateral mucosa from the same patients, and 65 healthy donors were evaluated for DNA methylation analyzing 18 target genes by quantitative bisulfite next-generation sequencing. We further evaluated an independent cohort (validation dataset) made of 20 normal donors, one oral fibroma, 14 oral lichen planus (OLP), three proliferative verrucous leukoplakia (PVL), and two OSCC.

**Results:**

Comparing OSCC with normal healthy donors and contralateral mucosa in 355 CpGs, we identified the following epigenetically altered genes: *ZAP70*, *ITGA4*, *KIF1A*, *PARP15*, *EPHX3*, *NTM*, *LRRTM1*, *FLI1*, *MIR193*, *LINC00599*, *PAX1*, and *MIR137HG* showing hypermethylation and *MIR296*, *TERT*, and *GP1BB* showing hypomethylation*.* The behavior of *ZAP70*, *GP1BB*, *H19*, *EPHX3*, and *MIR193* fluctuated among different interrogated CpGs. The gap between normal and OSCC samples remained mostly the same (Kruskal-Wallis *P* values < 0.05), but the absolute values changed conspicuously. ROC curve analysis identified the most informative CpGs, and we correctly stratified OSCC and HGSIL from normal donors using a multiclass linear discriminant analysis in a 13-gene panel (AUC 0.981). Only the OSCC with sarcomatoid features was negative. Three contralateral mucosa were positive, a sign of a possible field cancerization. Among imprinted genes, only *MIR296* showed loss of imprinting. *DNMT1*, *TERC*, and *H19* together with the global methylation of *long interspersed element 1* were unchanged. In the validation dataset, values over the threshold were detected in 2/2 OSCC, in 3/3 PVL, and in 2/14 OLP.

**Conclusions:**

Our data highlight the importance of CpG location and correct estimation of DNA methylation level for highly accurate early diagnosis of OSCC.

**Electronic supplementary material:**

The online version of this article (doi:10.1186/s13148-017-0386-7) contains supplementary material, which is available to authorized users.

## Background

Oral and pharyngeal cancer, grouped together, is the sixth most common cancer in the world. The annual estimated incidence is approximately 600,000 per year, two thirds of these cases occurring in developing countries [1]. The prevalence of oral cancers is high especially in South and Southeast Asia, where distinct cultural practices such as betel-quid chewing and varying patterns of tobacco and alcohol use are important risk factors that predispose to cancer of the oral cavity. The mortality rates of these tumors have remained unchanged (50% within 5 years after diagnosis) and are related to tobacco smoking and alcohol intake. Oral cancer patients are usually diagnosed at an advanced stage (two thirds are III–IV), which is associated with a worse prognosis and higher radio- and chemotherapy morbidity. Moreover, quality of life is disproportionately compromised in the oral cavity patient since surgical therapy can be mutilating and often has significant effects on swallowing, speech, and physical appearance. Evidently, improved oral cancer prevention, early detection, and better diagnostic and clinical management tools are needed to identify high-risk patients, such as those with smoking and alcohol exposure, patients without adequate access to health care, and patients with high-risk lesions such as oral leukoplakia (OL), which may progress to carcinoma. The estimated prevalence of OL is approximately 0.5% worldwide. Oral squamous cell carcinoma (OSCC) is the most common neoplastic disease in the head and neck region and is commonly preceded by oral premalignant lesions (OPML) such as OL. In Western countries, the annual malignant transformation rate from OL to OSCC varies from 0.13 to 36.4% [2, 3].

In addition, OSCC patients can develop a second primary OSCC, with a frequency ranging between 17 and 30% [4]. Clinical and histological features of OPML do not provide enough information to identify premalignant lesions at high risk of malignant transformation or patients who will develop an OSCC during follow-up. OSCC or high-grade squamous intraepithelial lesions (HGSIL) are usually diagnosed by incisional biopsy. Nevertheless, the biopsy requires a minimally invasive surgical approach that can create discomfort and may be refused by the patient. Moreover, only one third of patients present with early stage disease (T1–2, N0), and for the remainder, life expectancy is very short. Different researchers, including our group, have recently proposed an attractive strategy to reduce the burden of OSCC by analyzing the methylation status of a panel of genes starting from saliva and/or brushing specimens [5–10]. In many cancers, gene silencing by promoter methylation seems to be an early event in carcinogenesis and may occur even more frequently than structural inactivation of genes by mutations and deletions [11]. Histologically normal tissue adjacent to tumors and OPML can have an aberrant methylation pattern in candidate genes, suggesting that these epigenetic modifications are early events in oral carcinogenesis [12]. This study assessed the methylation status of a set of 18 gene promoters and *long interspersed element 1* (*LINE1*) to detect early OSCC and OPML by bisulfite next-generation sequencing (NGS), starting from minimal invasive collection specimens obtained by oral brushing.

## Methods

### Clinical samples

#### Ethics statement

All clinical investigations were conducted according to the principles of the Declaration of Helsinki. The study was approved by the local ethics committee (study number 14092, protocol number 899/CE). All information regarding the human material used in this study was managed using anonymous numerical codes.

#### Training dataset

We enrolled 28 consecutive patients with a clinical and histological diagnosis of OSCC, one case of OSCC with sarcomatoid features, six consecutive patients with a clinical and histological diagnosis of HGSIL as defined by Gale et al. [13, 14], 35 related contralateral clinically normal mucosa, and 65 normal mucosa of healthy donors as reference controls. Clinical data including smoking status are summarized in Table 1, and all these cases referred to the Department of Oral Sciences, University of Bologna, from January 2014 to December 2016. All patients presenting with a suspected oral neoplastic lesion that required diagnostic incisional biopsy also underwent oral brushing sampling. Lesions with an obvious etiology such as trauma and infective aphthous ulcerations were excluded. Oral brushing for histological diagnosis was performed as previously reported [15]. Specimens were always selected before incisional biopsy and samples were enrolled in the population study only after histological confirmation of OSCC. Two different brushing specimens were collected in OSCC and HGSIL patients: one from the area with a lesion and the other from clinically normal distant mucosa (cheek opposite).

**Table 1:** Clinical features of study population

	Number	Sex	Median age	Smoke	Site	*T* status	*N* status
Training dataset							
OSCC	29	14 males 15 females	69 ± 13	21 non-smokers 8 smokers	8 tongue 4 floor of mouth 8 gum 5 cheek 3 palate 1 lip	11 T1 10 T2 2 T3 6 T4	28 N0 1 N1
HGSIL	6	5 males 1 females	63 ± 18	6 non-smokers	6 tongue		
Healthy donors	65	32 males 33 females	58 ± 18	57 non-smokers 8 smokers	18 tongue 10 floor of mouth 12 gum 20 cheek 5 palate		
Validation dataset							
Healthy donors	20	10 males 10 females	45 ± 9	16 non-smokers 4 smokers	20 tongue + cheek		
OSCC	2	1 male 1 female	61 ± 4	2 non-smokers	1 tongue 1 hard palate	1 T2 1 T1	1 N1 1 N0
PVL	3	2 male 1 female	57 ± 6	2 non-smokers 1 smoker	2 tongue 1 cheek		
OLP	14	10 male 4 female	59 ± 6	11 non-smokers 3 smokers	12 cheek 2 gum		

**Fig. 1:**
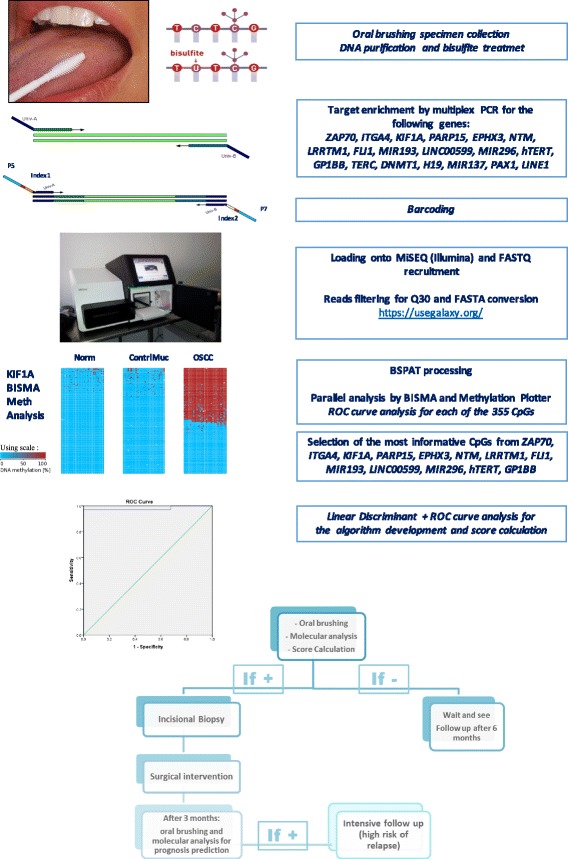
Assay flow chart: the assay design is based on various steps including minimal invasive collection of specimens by simple oral brushing in the suspected area. DNA purification and bisulfite treatment (unmethylated cytosines are chemically converted to uracyls, while methylated cytosines remained unchanged). Target-specific amplification of a set of 18 genes and *LINE1* with primers of choice. Barcoding using Nextera™ index kit (Illumina), pooling and loading onto MiSEQ. Quality control of FASTQ files and filtering for > Q30 and > 80 bp in length. FASTQ to FASTA conversion and loading onto BSPAT for mapping and methylation level evaluation; parallel evaluation using perl followed by BISMA and Methylation plotter. ROC curve analysis of each of the 355 CpGs. Identification of the most informative CpGs from the following genes: *ZAP70*, *ITGA4*, *KIF1A*, *PARP15*, *EPHX3*, *NTM*, *LRRTM1*, *FLI1*, *MIR193*, *LINC00599*, *MIR296*, *TERT*, and *GP1BB*. An algorithm of choice was then created taking into account all of the most informative CpGs from a panel of 13 genes, using linear discriminant analysis followed by a ROC curve to calculate the exact threshold able to discriminate OSCC from normal samples. In case of a positive score, the patient follows conventional treatment with incisional biopsy and surgical intervention. After 3 months, we propose evaluating the area around the surgical intervention to identify any field cancerization and an associated high risk of recurrence

Histological examination was performed blindly by two pathologists (MPF, SA) at the Department of Biomedical and Neuromotor Sciences, “M. Malpighi” Section of Anatomic Pathology, at Bellaria Hospital, University of Bologna, Italy. A multihead microscope discussion was made on discordant cases to obtain a common diagnosis. Histological diagnoses were established following the WHO criteria [14, 16] and according to the 2014 Ljubljana classification [17]. The oral brushing sample series included a group of 65 normal mucosa samples collected from healthy donors as normal controls. Only one oral brushing sample was collected in this group from similar areas with respect to the OSCC group. The flow chart in Fig. 1 depicts the experimental design of the study.

#### Validation dataset

We validate our results by an independent cohort of cases including 20 normal donors, one oral fibroma, 14 oral lichen planus, three proliferative verrucous leukoplakia, and two OSCC enrolled from January to May 2017.

### DNA purification and bisulfite treatment

DNA from exfoliating brush specimens was purified using *The MasterPure*™ Complete *DNA* Purification Kit (Epicentre, cod. MC85200). Bisulfite treatment of genomic DNA (200–500 ng) was carried out with the *EZ* DNA Methylation-Lightning™ Kit (Zymo Research, cod. D5031) according to the manufacturer’s protocol.

### Gene selection

A set of 19 gene targets were selected because they were previously identified with altered methylation pattern in OSCC. A detailed list of reference for each gene is available in Table 2. In particular, *ZAP70*, *KIF1A*, *GP1BB*, and *MIR137HG* were previously described to be differentially methylated in OSCC by our group [15] and others [5, 18, 19], while *PAX1*, *LRRTM1*, *PARP15*, *FLI1*, *NTM*, *LINC00599*, *EPHX3*, and *ITGA4* were discovered by Guerrero-Preston et al. [20]. Additionally, the remaining *MIR193A*, *MIR296*, *TERT*, *TERC*, *H19*, *DNMT1A*, and *LINE1* were found to be epigenetically altered in OSCC by various authors [21–27] (see Table 2 for details).

**Table 2:** List of genes interrogated in this study, mapping information, coordinates, and imprinting status

Gene	Description	Reference	Map	RefSEQ	Position	UCSC h19 coordinates	Amplicon length	Position with respect to the TSS	Number of interrogated CpG	Imprinting status
*ZAP70*	Zeta chain of T cell receptor-associated protein kinase 70	15; 19	2q11.2	NM_001079	Exon 3	Chr2 98340750–98340885	180	10,728	20	No
*GP1BB*	Glycoprotein Ib platelet beta subunit	15; 19	22q11.21	NM_000407	Exon 1	Chr22: 19710829–19710960	192	363	18	No
*KIF1A*	Kinesin family member 1A	15; 5	2q37.3	NM_001244008	Exon 1	Chr2: 241759586–241759727	189	−1	27	No
*PARP15*	Poly(ADP-ribose) polymerase family member 15	20	3q21.1	NM_001113523	Exon 1	Chr3:122296564–122296725	206	93	19	No
*ITGA4*	Integrin subunit alpha 4	20	2q31.3	NM_000885	Exon 2	Chr2: 182322887–182323053	214	912	14	No
*NTM*	Neurotrimin	20	11q25	NM_016522	Exon 1	Chr11: 131781042–131781185	190	62	15	Yes, maternal, No SNP
*MIR193A*	MicroRNA 193a	18; 60	17q11.2	NR_029710	Promoter	Chr17: 29886860–29887068	256	−178	26	No
*MIR137HG*	MIR137 host gene7	15; 19; 41	1p21.3	NR_046105	Exon 1	Chr1: 98511645–98511814	216	136	16	No
*EPHX3*	Epoxide hydrolase 3	20	19p13.12	NM_024794	Exon 1	Chr19: 15342826–15343049	223	215	29	No
*LINC00599*	Long intergenic non-protein coding RNA 599	20	8p23.1	NR_029668	Exon 1	Chr8: 9760739–9760890	199	69	20	No
*FLI1*	Fli-1 proto-oncogene, ETS transcription factor	20	11q24.3	NM_001271010	Exon 1	Chr11: 128564020–128564160	186	187	12	No
*MIR296*	MicroRNA 296	20; 51	20q13.32	NR_029844	Exon 1	Chr20: 57392355–57392545	238	180	15	Yes, paternal, No SNP
*LRRTM1*	Leucine-rich repeat transmembrane neuronal 1	20	2p12	NM_178839	Promoter	Chr2: 80531676–80531807	179	−431	24	Yes, paternal, No SNP
*TERT*	Telomerase reverse transcriptase	53	5p15.33	**NM_198253**	Intron4–5	Chr5: 1279743–1279851	109	14,976	6	No, SNP at rs10069690
*DNMT1*	DNA methyltransferase 1	26	19p13.2	NG_028016.3	Exon 1	Chr19: 10305498–10305665	216	142	28	Yes, paternal, No SNP
*TERC*	Telomerase RNA component	22	3q26.1	NG_016363.1	Exon 1	Chr3: 169764557–169764699	191	357	19	No
*PAX1*	Paired box 1	20	20p11.23	NM_001257096	Exon 1	Chr20: 21705602–21705713	159	−60	13	No
*H19*	H19 conserved region 1	24	11p15.4	NR_002196	Exon 1	Chr11: 1996891–1997062	219	159	13	Yes, maternal, SNP rs2067051
*LINE1*	Long interspersed element 1	36; 25	Wide	M80343.1	Exon 1	Genome-wide distribution	267	458	21	No

### In silico prediction of CpG island and primer design

To identify the putative CpG island on the promoter region or in the first part of the coding region, the genomic sequence stored on the Ensembl genome browser (http://www.ensembl.org/index.html) including 1000 bp upstream of the ATG site were employed as query sequence. MethPrimer (http://www.urogene.org/cgi-bin/methprimer/methprimer.cgi) [28] designing was applied to identify CpGs and the best primers of choice. Regions of interest and mapping coordinates are listed in Table 2.

### NGS library preparation

The library was prepared in two steps: a first multiplex PCR amplification for target enrichment and a second round of amplification with a low number of cycles allowing the barcoding of the template-specific amplicons obtained from the first amplification step. Barcoding was performed using the Nextera™ index kit as previously described [29]. Locus-specific bisulfite amplicon libraries were generated with tagged primers using *Phusion U* DNA polymerase (ThermoFisher, cod. F555L).

Amplification products were purified using SPRI-AMPure XT (Agencourt-Beckman Coulter, cod. A63881) quantified with Fluorometer Quantus™ (Promega, cod. E6150) and then employed as template (100 ng) for a second round of PCR (6 cycles). Sample-specific barcode sequences were added in this second PCR. The amplicon library was purified using Agencourt AMPure XP beads (Agencourt-Beckman Coulter, cod. A63881) and then quantitated with the Quantus™ Fluorometer (Promega, cod. E6150). Sequencing was conducted on the MiSEQ (Illumina, cod. 15027617) according to the manufacturer’s protocol. A set of three genes *KIF1A*, *ZAP70*, and *GP1BB* were initially evaluated in parallel by the protocol described by Morandi et al. [15] using pyrosequencing on GSJunior (Roche) to verify the quantification method in a set of 10 normal donors.

### NGS data analysis

FASTQ files already trimmed for multiplex identifier were processed for quality control (> Q30) and for read lengths (> 100 bp) and converted into FASTA format in a Galaxy Project environment [30]. To evaluate the methylation ratio of each CpG, we loaded FASTA files into the bisulfite sequencing pattern analysis tool (BSPAT—http://cbc.case.edu/BSPAT/index.jsp) [31]. In parallel, we used Perl to create single specific files for every interrogated gene, which were then visualized using BiQ Analyzer [32], QUMA [33], and BISMA [34] to confirm data from BSPAT analysis. Methylation plotter (http://gattaca.imppc.org:3838/methylation_plotter/) [35] was used to create Fig. 4 and Additional file 1.

### Statistical analysis

Multiclass linear discriminant analysis and receiver operating characteristic (ROC) curve analysis were calculated using IBM SPSS Statistics 21 (IBM) and the easyROC web tool (http://www.biosoft.hacettepe.edu.tr/easyROC/). PCA analysis and the methylation plot were created using ClustVis, a web tool for visualizing clustering multivariate data (http://biit.cs.ut.ee/clustvis/) [36].

## Results

### Methylation analysis

Bisulfite NGS was used to examine the set of 18 genes listed in Table 2 together with global methylation analysis evaluating *long interspersed element 1* (*LINE1*), with a total of 355 CpGs, mostly located within the first exon.

Parallel evaluation using our new bisulfite NGS method on Illumina MiSEQ and the one described by Morandi et al. [15] using pyrosequencing on GSJunior (Roche) was performed in a set of 10 normal donors for *KIF1A*, *GP1BB*, and *ZAP70* giving almost identical results (Spearman correlation of 0.969). A further index of reliability of the assay is the *H19* mean value of 13 CpGs evaluated in normal donors, which was found very close to 50% as expected: 0.47 ± 0.14 (see Additional file 2).

**Fig. 2:**
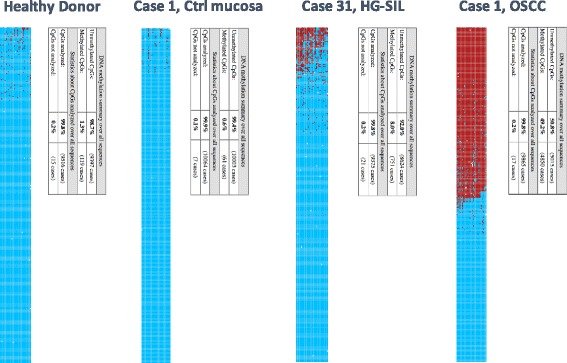
Graphic representation of methylation analysis for *KIF1A* among a normal healthy donor, contralateral mucosa and OSCC from case 1, and HGSIL case 31. Unmethylated CpGs are in *blue*, while methylated CpGs are in *red*. A single table for each specimen summarized the whole methylation frequency for the 27 CpGs tested

An example of the methylation analysis using BISMA (http://services.ibc.uni-stuttgart.de/BDPC/BISMA/manual_unique.php) [34] as a web tool is shown in Fig. 2, representing a comparison for *KIF1A* among case 1 (OSCC), its normal contralateral mucosa, case 31 (HG-SIL), and a healthy donor. Most OSCC cases had a homogeneous methylation pattern independently of the ratio, while all six HGSIL enrolled in this study showed an irregular methylation pattern among various CpGs as exemplified in Fig. 2 for case 31.

**Fig. 3:**
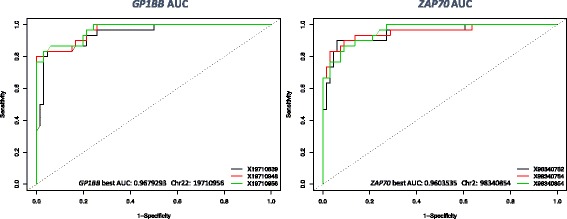
ROC analysis discriminating OSCC vs normal healthy donors using easyROC as a webtool, showing the three best performing CpGs from *GP1BB* and *ZAP70*. Comparing OSCC vs normal healthy donors, these two genes revealed the highest discrimination power: *ZAP70* showing hypermethylation and *GP1BB* showing hypomethylation*.* ROC analysis of all of the gene targets evaluated in this study is available in Additional file 3

After BSPAT data processing [31] (see “Methods” for the bioinformatic pipeline), a ROC analysis for each CpG of all 18 genes evaluating differences between OSCC and normal healthy donors is performed and summarized in Additional file 3 (only the three best CpGs were shown for simplicity). In Fig. 3, the ROC of the most performant genes *GP1BB* and *ZAP70* were displayed. *DNMT1*, *TERC*, and *H19* were found to be unchanged both evaluating OSCC vs normal donors and vs normal contralateral mucosa.

**Fig. 4:**
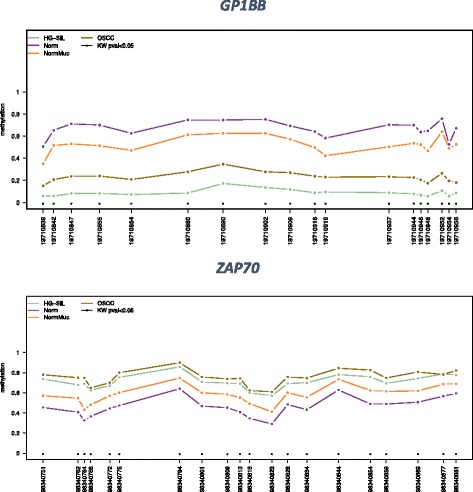
Methylation profile plot from *GP1BB* and *ZAP70*. For each group of samples, each line represents the methylation mean for each position. *Asterisks* indicate a statistical significance as calculated by the Kruskal-Wallis test*. ZAP70* and *GP1BB*, together with *H19*, *EPHX3*, and *MIR193* (see Additional file 1 for the methylation profile of all targets), revealed a fluctuating behavior among the various CpGs evaluated. The gap between normal and OSCC remained mostly the same (Kruskal-Wallis *P* values were < 0.05), but the absolute values changed conspicuously among different positions investigated

Among the remaining 15 differentially methylated genes, 12 were hypermethylated (*ZAP70*, *PAX1*, *KIF1A*, *LRRTM1*, *PARP15*, *FLI1*, *NTM*, *LINC0059*, *EPHX3*, *MIR137HG*, *ITGA4*, *MIR193*), whereas three were hypomethylated (*GP1BB*, *MIR296*, *TERT*) in OSCC/HGSIL. Additional file 1 depicts the mean methylation level for each group of the interrogated genes visualized using the Methylation plotter tool [35] and the Kruskal-Wallis test for single CpG. Figure 4 shows the fluctuating methylation level among different CpGs evaluated in *GP1BB* and *ZAP70*. Mean methylation level and corresponding standard deviation of each group for all target genes is available in Additional file 2.

Considering only one gene at a time, the AUC varied depending on the various CpGs involved; ranges are summarized in Table 3 for the best and worst AUC values.

**Table 3:** The best and worst AUC values among the 18 genes evaluated in this study. *KIF1A* showed a minimal variation among all 27 CpGs interrogated, whereas *TERT* displayed the widest gap between different AUC, highlighting the importance of CpG location

Gene	Best AUC position	Best AUC value	Worst AUC position	Worst AUC value	Best to worst AUC gap
*KIF1A*	Chr2: 241759621	0.94	Chr2: 241759702	0.87	0.0694444
*MIR137*	Chr1: 98046097	0.78	Chr1: 98046237	0.69	0.0916666
*GP1BB*	Chr22: 19710956	0.97	Chr22: 19710954	0.88	0.0921717
*H19*	Chr11: 2018169	0.66	Chr11: 2018228	0.56	0.0992425
*LRRTM1*	Chr2: 80531799	0.9	Chr2: 80531776	0.8	0.1012626
*NTM*	Chr11: 131781167	0.86	Chr11: 131781080	0.75	0.1063131
*ZAP70*	Chr2: 98340854	0.96	Chr2: 98340816	0.85	0.1073232
*MIR193A*	Chr17: 29886944	0.84	Chr17: 29886881	0.72	0.1252525
*EPHX3*	Chr19: 15342885	0.83	Chr19: 15342831	0.71	0.1260101
*LINC00599*	Chr8: 9760888	0.83	Chr8: 9760805	0.69	0.1436869
*FLI1*	Chr11: 128564158	0.84	Chr11: 128564065	0.67	0.1643940
*PAX1*	Chr20: 21686312	0.9	Chr20: 21686253	0.74	0.1648990
*ITGA4*	Chr2: 1823229.02	0.84	Chr2: 182323028	0.66	0.1813131
*PARP15*	Chr3: 122296586	0.84	Chr3: 122296671	0.65	0.1919191
*TERT*	Chr5: 1279758	0.92	Chr5: 1279838	0.67	0.2563132
*MIR296*	Chr20: 57392374	0.75	Chr20: 57392419	0.44	0.3151516
*DNMT1*	Chr19: 10305597	0.67	Chr19: 10305652	0.34	0.3338384
*TERC*	Chr3: 169482446	0.69	Chr3: 169482370	0.28	0.4047980

### Algorithm development

The best CpGs identified by the ROC analysis were used to create an algorithm of choice to correctly discriminate OSCC and HGSIL, with the exception of *MIR137HG*, which was previously shown to be aberrantly methylated also in oral lichen planus (OLP) [15], and *PAX1* which was used as an informative marker for a patent related to prognosis prediction in Head and Neck Squamous Cell Carcinoma (HNSCC) [20]. A multiclass linear discriminant analysis (LDA) that weighted the contribution of each CpG was used to calculate the score.

**Fig. 5:**
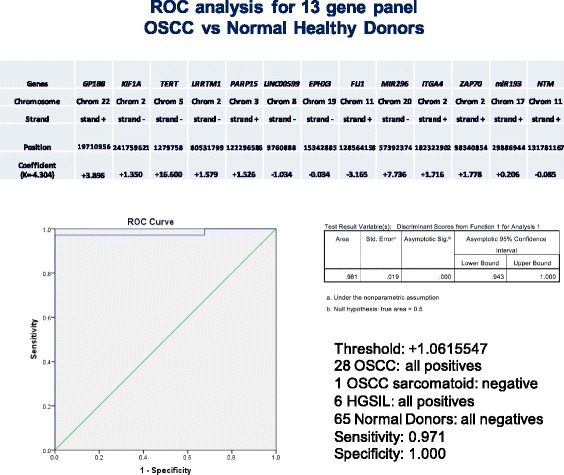
ROC curve analysis discriminating OSCC vs normal healthy donors using the score calculated form the algorithm. AUC detected is 0.981. The best informative CpGs used in this new 13-gene algorithm and their values are shown in each column with chromosome number and mapping information

ROC curve analysis applied to these scores discriminating OSCC and HGSIL from normal donors gave an area under the curve (AUC) of 0.981, identifying a threshold of 1.0615547 as the best value for sensitivity and specificity (97.1 and 100% respectively, see Fig. 5). Twenty-eight out of 29 OSCC (96.6%) and six out of six HGSIL (100%) specimens exceeded the threshold value. The squamous cell carcinoma with sarcomatoid features was the only OSCC case found to be negative for the score with no sign of epigenetic changes in any of the genes evaluated. None of the 65 healthy donor specimens showed a positive result, and three out of 30 available contralateral normal mucosa specimens from OSCC patients exceeded the threshold value (10%). Five cases of contralateral mucosa did not yield enough DNA to perform the test, probably for inadequate repeat brushings.

### Kruskal-Wallis test and multiple range test analyses

**Fig. 6:**
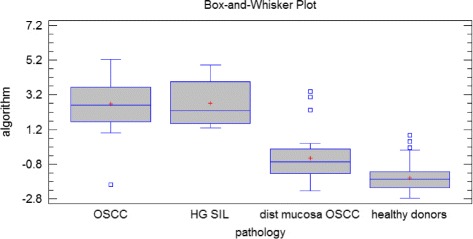
Box plots obtained using the scores calculated from the algorithm showed a between-group significant difference (Kruskal-Wallis test *T* = 78.8587, *P* < 0.05)

Most of the investigated CpGs had a Kruskal-Wallis *P* value < 0.05 (Fig. 4). Kruskal-Wallis test showed a significant between-group difference for scores generated with linear discriminant analysis (LDA) (*T* = 78.8587, *P* < 0.05) (Fig. 6). Multiple range test for LDA-generated scores did not show a statistical difference between the OSCC and HGSIL groups, whereas it identified a statistical difference between the OSCC group and healthy donors and contralateral mucosa. Furthermore, multiple range test showed a statistical difference between the HGSIL group and healthy donors and contralateral mucosa. Multiple range test identified a statistical difference between healthy donors and contralateral mucosa (see Table 4 for details).

**Table 4:** Multiple range test did not show significant differences in the generated score between OSCC group and HGSIL group. Both groups showed significant higher values whether considering healthy donors or considering contralateral mucosa. Multiple range test showed a statistical difference between contralateral mucosa of OSCC and HGSIL patients and healthy donors

Method: 95.0% LSD			
Pathology	Count	Mean	Homogeneous groups
Healthy donors	65	−1.586	C
Distant mucosa in OSCC patients	30	−0.444399	B
OSCC	29	2.66856	A
HGSIL	6	2.71892	A
Contrast	Sig.	Difference	± limits
OSCC–HGSIL		−0.0503633	0.983538
OSCC–distant mucosa in OSCC patients	*	3.11296	0.571081
OSCC–healthy donors	*	4.25456	0.489711
HGSIL–distant mucosa in OSCC patients	*	3.16332	0.980724
HGSIL–healthy donors	*	4.30492	0.935683
Distant mucosa in OSCC patients–healthy donors	*	1.1416	0.484034

*Statistically significant difference

### Imprinted gene evaluation

Among the 18 genes tested, *DNMT1*, *MIR296*, *NTM*, *LRRTM1*, and *H19* were previously considered to be imprinted (see geneimprint database, http://www.geneimprint.com/site/genes-by-species). In this study, *DNMT1*, *NTM*, and *LRRTM1* showed very low levels or absence of methylation in normal donors and in contralateral mucosa. We further analyzed DNA from whole blood DNA of a pool of healthy female donors (DNA female pool, Cod. G1521, Promega, Madison, WI, USA, data not shown) with absence of methylation as confirmation. On the contrary, *H19* showed a classical imprinted status with 50% methylation both in normal mucosa and in different lesions. No evidence of an altered methylation pattern in OSCC cases was found with a mean methylation value of 0.56 ± 0.25 SD. A single CpG (Chr11: 2018169) revealed lower values (mean 0.30 ± 0.26 SD). The fluctuating behavior of the 12 CpGs in *H19* is shown in Additional file 1.

For *MIR296*, full methylation was discovered (mean value of 0.98 ± 0.02) in normal cells. This gene was hypomethylated in all but four cases of OSCC considering the most informative CpG mapped at Chr20: 57392374 (mean value of 0.95 ± 0.05). A detailed pattern of methylation for each CpG of this gene is shown in Additional files 1 and 3.

### PCA and heatmap

**Fig. 7:**
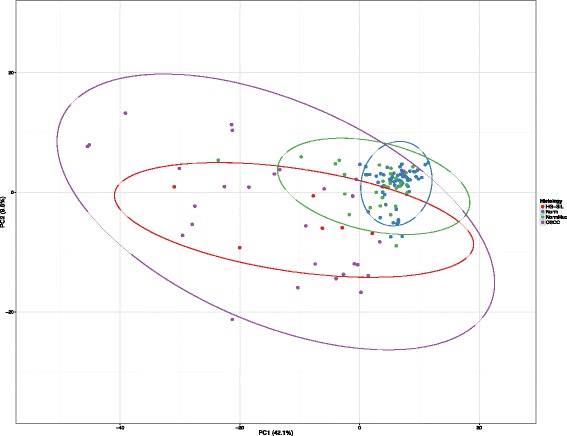
Principal component analysis (PCA): Unit variance scaling is applied to rows; SVD with imputation is used to calculate the principal components. *X* and *Y* axes show principal component 1 and principal component 2 that explain 42.1 and 9.6% of the total variance, respectively. Prediction ellipses are such that with probability 0.95, a new observation from the same group will fall inside the ellipse. *N* = 130 data points

Using the principal component analysis with the highest distribution of data (PC1) as the *x*-axis and the second highest principal component (PC2) as the *y*-axis, the data are distributed as evenly across the plot as possible while maintaining the distance between points as a proxy for how similar each point is to the other (Fig. 7). The graph shows that OSCC elements (violet) and HGSIL (red) are located in the left center of the plot, while normal donors (blue) are clustered in a well-defined and restricted area near the normal contralateral specimens (green) which span in a less defined manner.

**Fig. 8:**
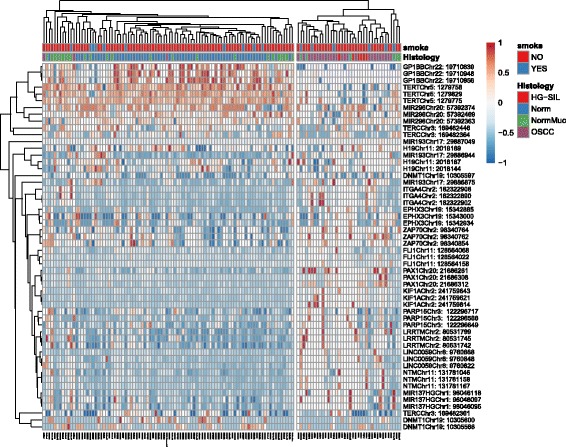
Heatmap from the best three CpG methylation data points (54 rows) for each gene and 130 samples (column). Annotation labels refer to histology and smoking status. Rows are centered; unit variance scaling is applied to rows. Both rows and columns are clustered using correlation distance and average linkage. Smoking status and histology are *highlighted in color*. Two clusters are marked: left cluster: 65 normal donors, 24 contralateral mucosa, two OSCC, and the OSCC with sarcomatoid features; right cluster: 26 OSCC, six HGSIL, and six contralateral mucosa. Complete heatmap from each of the 325 CpGs is available in Additional file 4

Figure 8 shows a heatmap based on the best three CpGs of the 18 genes evaluated. Values in the matrix are color-coded, and rows (CpGs) and columns (specimens) are clustered using correlation distance and average linkage. The top of the heatmap shows an overview of two annotations, histology and smoking status. Two clusters are marked: left cluster: 65 normal donors, 24 contralateral mucosa, two OSCC, and the OSCC with sarcomatoid features; right cluster: 26 OSCC, six HGSIL, and six contralateral mucosa. Complete heatmap from all CpGs is available in Additional file 4.

### LINE1 *global methylation*

*LINE1* methylation analysis was evaluated to interrogate global methylation status of repetitive elements distributed genome-wide [37]. As seen in Additional file 5, no differences among groups and no signs of hypomethylation were detected in OSCC.

### Algorithm validation in an independent cohort

Considering the validation dataset, all normal donors were detected under the threshold value, as well as one oral fibroma and 12 out of 14 OLP; on the contrary, the remaining two OLP, all PVL, and all OSCC were positives. PCA is available in Additional file 6 and heatmap in Additional file 7.

## Discussion

Screening populations for the early detection of asymptomatic carcinoma or precursor lesions is an attractive strategy to reduce the burden of OSCC. A major example of this approach is the cytological smear test in women for cervical cancer screening. However, low sensitivity and specificity have precluded the widescale adoption of microscopic cytology for the detection of oral cancer and OPML [38]. Methods such as toluidine blue staining and autofluorescence imaging have been proposed to improve the clinical identification of high-risk OPML, but a recent meta-analysis confirmed there was no evidence to support the use of these adjunctive technologies as screening tools to reduce oral cancer mortality [39]. Recent findings indicated that DNA methylation analysis of a specific set of genes may serve to detect early stage oral cancer lesions [22, 40], while the location of core regions and the density of methylation are required for gene silencing [41].

This study adopted a different approach from 450k methylation arrays to interrogate a set of genes already known to be altered in HNSCC [5, 15, 19, 20, 27]. For in-depth investigation of the true methylation level, we developed a novel accurate, sensitive, and specific assay to detect early OSCC and HGSIL from oral brushing specimens using bisulfite NGS. Quantitative DNA methylation analysis of the following 13 genes: *ZAP70*, *ITGA4*, *KIF1A*, *PARP15*, *EPHX3*, *NTM*, *LRRTM1*, *FLI1*, *MIR193*, *LINC00599*, *MIR296*, *TERT*, and *GP1BB*, clearly discriminated OSCC and HGSIL from healthy donors or from contralateral normal mucosa from the same patients. In particular, we found in OSCC and HGSIL hypermethylation of *ZAP70*, *ITGA4*, *KIF1A*, *PARP15*, *EPHX3*, *NTM*, *LRRTM1*, *FLI1*, *MIR193*, *LINC00599*, *PAX1*, and *MIR137HG* and hypomethylation of *TERT*, *MIR296*, and *GP1BB* at various CpGs. No epigenetic changes were found in *DNMT1*, *H19*, *TERC*, or any global hypomethylation of repetitive *LINE1.* Best performances were found for *ZAP70*, *GP1BB*, *PAX1*, *LRRTM1*, *KIF1A*, and *TERT* showing the best sensitivity and specificity in a ROC curve analysis (see the best CpGs for each gene in Figs. 3, 4, 5, and 6). Despite its high discriminant potential, we excluded the new panel *PAX1* described by Guerrero-Preston [20] because it was patented by the same group, and *MIR137HG* which yielded ambiguous results (see Additional files 1 and 3) and was previously found to be differentially methylated in OLP [15, 42].

The stratification among distinct groups (OSCC vs healthy normal individuals, OSCC vs normal contralateral mucosa) was based not simply on evaluation of the methylated/unmethylated allele but also on quantitative identification of the correct threshold among various CpGs of the different genes considered. Combining the methylation level of 13 genes, the discrimination power of this new assay reached an optimal level of accuracy (AUC 0.981). The only OSCC case negative for the score was the OSCC with sarcomatoid features. No aberrant methylation pattern was found in any of the genes investigated, signifying that this rare variant of oral carcinoma does not share the same epigenetic status as the other OSCC cases, as shown in Fig. 8 in cluster analysis. This result may be considered for clinical management, and further investigation into genomic alterations is required.

Linear discriminant analysis allowed us to generate a score that weighted the best CpGs from the most informative genes investigated in the study. A single gene discriminating method or a single CpG may be inadequate for our purpose. Considering only one gene at a time such as *GP1BB*, the AUC varied between 0.968 and 0.876 depending on the CpGs, while for *TERT*, it varied from 0.924 to 0.667, implying the central role of CpG location (see Table 3 for the best and worst AUC values).

By using an independent cohort of samples, we further validate our algorithm confirming a clear negative value for all normal donors enrolled, together with one oral fibroma and 12 OLP. On the contrary, values over the threshold were detected in the two OSCC, in all three PVL, and in two OLP. Positivity for all PVL may be related to its nature of a progressive, multifocal, exophytic form of leukoplakia with high rates of malignant transformation. For two OLP positives (14.3%), the World Health Organization (WHO) has considered OLP as a premalignant lesion. The range of malignant transformation varies between 0 and 10% [43], which is consistent with our findings.

Basically, the calculated thresholds of most interrogated CpGs were proximal to 0 in the following genes: *FLI1*, *ITGA4*, *KIF1A*, *MIR193*, *NTM*, *LINC00599*, *PAX1*, and *MIR137.* Overall, the aberrant methylation pattern of these OSCC-related markers was estimated to be 0.33 ± 0.3 SD, simply overcoming the basal unmethylated condition seen in normal cells from both the contralateral mucosa of the same patients and the oral mucosa of healthy donors. By contrast, *EPHX3*, *LRRTM1*, and *PARP15* showed higher values both for threshold and mean values for the OSCC group (0.61 ± 0.31 SD). This implies that the basal level of methylated CpG in these genes in normal tissues is not close to 0, and a small fraction of epialleles are methylated. Moreover, methylation levels in OSCC exceeded 50%. The behavior of a set of five genes including *ZAP70*, *GP1BB*, *H19*, *EPHX3*, and *MIR193* fluctuated among the various CpGs as shown in Additional file 1. Interestingly, the gap between normal and OSCC remained the same (Kruskal-Wallis *P* values were mostly < 0.05) but the absolute values changed conspicuously among different positions investigated. This implies that each CpG must be considered apart, and a specific threshold calculated. Recently, van Vlodrop et al. [41] emphasized the emerging evidence on the importance of the location of CpG methylation in relation to gene expression and associations with clinicopathologic characteristics in cancer. Crucial CpG islands or single CpGs for regulating gene expression not only are often situated around the transcriptional start site but can also be observed more upstream or downstream.

We used bisulfite NGS methods because of their advantages: (i) many CpG sites, so that complex methylation patterns of individual DNA molecules can be determined; (ii) the longer reads can be aligned to the reference sequence more easily and accurately, especially in repetitive regions of the genome; and (iii) the long reads are more likely to cover more genotype information like single nucleotide polymorphisms (SNPs) in the neighborhood of cytosines for correlation analysis between DNA methylation and genotype. These features identified not simply different methylation levels among groups but for instance a different pattern of methylation between OSCC and HGSIL. Usually, OSCC revealed a homogeneous pattern with most epialleles methylated *in cis*, whereas HGSIL disclosed an irregular pattern of CpG methylation as shown in Fig. 2 for case 31. This behavior was shared among all five HGSIL for all the genes involved and is probably due to partial epigenetic modifications which reach a complete pattern only in full-blown OSCC. This behavior is visible only using bisulfite NGS and not methylation-sensitive PCR or qPCR, or Infinium™. A previous report by Bock et al. [44] compared the most promising assays for measuring DNA methylation in large cohorts, clinical diagnostics, and biomarker development, including amplicon bisulfite sequencing, enrichment bisulfite sequencing, Infinium™, EpiTyper®, and Pyroseq™. Best performances were obtained using amplicon bisulfite sequencing with high accuracy and robustness. In addition, this approach guarantees high throughput and a variable number of targets to interrogate, depending on the assay design [45].

We did not identify *LINE1* global hypomethylation in OSCC with respect to the normal donors and normal contralateral mucosa as previously described [25] using pyrosequencing technique. With respect to the pyrosequencing approach which interrogates only four CpGs, as indicated by Ogino et al. [46], we evaluated more CpGs (21), with high number of reads per single specimen (hundreds). However, probably, we select a CpG-rich region which may not be involved in changes in methylation.

From a bioinformatic point of view, bisulfite NGS analysis is complex and the pipeline requires many steps. Commonly, NGS runs produce FASTQ files as output already trimmed for multiplex identifier or MID/IonXpress/Index (a short barcode sequence used to label samples/patients when multiplexing) to recognize loaded specimens. Firstly, these FASTQ files are processed for quality control (> Q30 for Illumina) and read lengths (> 100 bp) to discard primer dimers, then FASTQ files are converted to FASTA. With the advent of cloud computing and the availability of NGS webtools such as Galaxy Project [47], these steps have minimal PC requirements and are user friendly. Using perl to recognize specific sequence motifs, several FASTA files were created for each gene for each patient and then loaded onto a visualization tool such as BiQ Analyzer [48], MethVisual [49], QUMA [33], or BISMA [34]. However, these tools do not scale up with massively parallel sequencing having been designed for Sanger sequencing. Newer tools such as Bismark [50] and BS-Seeker [51] have been utilized more efficiently and can handle larger datasets generated by NGS technologies. In addition, a web application service named bisulfite sequencing pattern analysis tool (BSPAT) uses Bismark’s read alignments and methylation calling functionalities to provide further quality control, co-occurrence pattern analysis, simple allele-specific methylation analysis, visualization, and integration with other databases and tools [31]. BSPAT gives the correct methylation ratio of each epiallele investigated to be included in subsequent statistical analysis. We therefore used this tool to analyze our data as the best option, maintaining BISMA as a supporting tool for confirmation.

*NTM*, *LRRTM1*, *MIR296*, *H19*, and *DNMT1* are known to be imprinted genes and are included in the imprinting gene database (http://www.geneimprint.com). However, no normal healthy donors, contralateral normal mucosa or DNA from whole blood of a pool of healthy female donors was either hemimethylated or fully methylated in *DNMT1*, *LRRTM1*, and *NTM* in our series. By contrast, *H19* showed common signs of imprinting with half of the epialleles methylated in normal donors and contralateral normal mucosa. We found no evidence for an altered methylation pattern in OSCC cases with a mean methylation value of 0.56 ± 0.25 SD. For *MIR296*, another imprinted gene, a mean value of 0.95 ± 0.05 was discovered in normal donors, while it was found to be hypomethylated in all but two cases of OSCC (mean 0.91 ± 0.07, see Additional file 2). This is in agreement with a full methylation of both epialleles, while in lesions, we found a partial demethylation of only nine out of 15 CpGs tested (Additional file 1). Aberrant expression of *MIR296* was previously related to gastric [52], bladder [53], and lung cancer [54]. The same behavior was found for *TERT* which was fully methylated in both epialleles in normal samples and partially hypomethylated in OSCC/HGSIL. Hypomethylation of this gene was previously reported in glioblastoma [55, 56]. Five out of six CpGs of the *TERT* gene were informative, even if the discriminating gap among different classes was found to be very slight, but calculating the ROC curve, the AUC obtained was highly consistent (0.921, see Additional file 3 for ROC between OSCC and normal donors).

Among the other 11 genes included in our algorithm, Marsit et al. [19] and our group previously identified three (*GP1BB*, *ZAP70*, *KIF1A*) as possible markers [15]. The present study provided evidence for a complete demonstration of their real value with the best AUC in the ROC analysis.

*GP1BB* encodes heterodimeric transmembrane protein that constitutes the receptor for von Willebrand factor and mediates platelet adhesion in the arterial circulation. Mutations of *GP1BB* are associated with Bernard-Soulier syndrome, an extremely rare inherited bleeding disorder [57]. *ZAP70* gene encodes the ζ-chain associated protein kinase 70 kDa, which is a tyrosine kinase normally expressed by natural killer cells and T cells. Hypermethylation of *ZAP70* gene predicted an unfavorable disease course in terms of disease progression and overall survival in chronic lymphocytic leukemia [58]. *KIF1A* (kinesin family member 1A) encodes a protein that is microtubule-dependent molecular motor involved in important intracellular functions such as organelle transport and cell division [59].

The other eight markers identified here included *PAX1* and *PARP15* already described by Guerrero-Preston et al. [20] as related to oral cancer, *FLI1* involved in Ewing sarcoma [60], rectal cancer [61], and gastric cancer [62], *NTM* in prostate [63], *EPHX3* in salivary gland adenoid cystic carcinoma [64], *ITGA4* in colorectal cancer [65] ,and *MIR193* in gastric cancer [66].

A second interesting finding of the present study was the behavior of normal distant mucosa from OSCC patients. Kruskal-Wallis test and multiple range test showed that the mean methylation value obtained in the group of normal distant mucosa of OSCC patients was significantly (*P* < 0.01) lower than the methylation value of the OSCC area but, at the same time, significantly higher (*P* < 0.01) than the methylation value of normal mucosa from healthy donors. Furthermore, three out of 30 normal distant mucosa (10%) exceeded the threshold, and this behavior in our opinion may not be due to a possible contamination of few cancer cells during the collection of normal mucosa, since our algorithm is based on quantitative methylation analysis and not simply on methylated/unmethylated status.

Genetic and epigenetic changes are also detected in histopathologically clean resection fields and could cause local relapse in mucosa primarily free of cancer cells. This has been explained by Slaughter’s model of “field cancerization” [67], whereas Braakhuis et al. [68] proposed a “patch-field” model in which a stem cell acquires genetic and epigenetic alterations in the initial phase, forming a clonal unit of altered daughter cells called a “patch.” A patch will transform into an expanding field acquiring additional genetic alterations, and by virtue of its growth advantage, a proliferating field gradually displaces the normal mucosa. Assuming that mucosa is predisposed to carcinogenesis due to exposure to exogenous genotoxins, an important clinical implication is that fields often remain after surgery of the primary tumor and may lead to new cancers clinicians currently refer to as “a second primary tumor” or “local recurrence,” depending on the exact site and time interval. The areas at highest risk for development of a second squamous cell carcinoma are large, sometimes extending to the lung [69]. Genetically altered cells may escape macroscopic or histopathological examination and may require sophisticated biomolecular approaches. An altered pattern of gene methylation in morphologically normal mucosa in OSCC patients may indicate field cancerization, so that these patients could have a higher risk of developing a second primary tumor or local recurrence. Further study on the methylation pattern in surgically resected patients may expand the potential of our new assay even for prognostic applications.

## Conclusions

In conclusion, the present study confirmed the role of epigenetic alterations and aberrant methylation DNA status in HGSIL/OSCC and also revealed an altered methylation pattern in normal mucosa distant from the OSCC area. Early diagnosis of OSCC may be important for clinical management, particularly in high-risk populations, and our novel assay based on quantitative bisulfite NGS analysis could be a highly sensitive and specific method to detect early OSCC starting from non-invasive, easy-to-perform brush sampling. Further studies with a larger cohort including more HGSIL, low-grade SIL, and OLP with 5 years of follow-up are needed to elucidate the intrinsic prognostic potential of our assay.

## Additional files

Additional file 1: Methylation profile plot from 18 genes evaluated. For each group of samples, each line represents the methylation mean for each position. Asterisks indicate a statistical significance as calculated by the Kruskal-Wallis test*. ZAP70*, *GP1BB*, *H19*, *EPHX3*, and *MIR193* revealed a fluctuating behavior among the various CpGs evaluated. The gap between normal and OSCC remained mostly the same (Kruskal-Wallis *P* values were < 0.05), but the absolute values changed conspicuously among different positions investigated. (PDF 415 kb)

Additional file 2: Summary tables of each gene targets with the mean, the standard deviation, the minimum, the maximum, and the number of missing data for each position and group of samples. This test is the non-parametric version of the ANOVA (one-way analysis of variance) and tests whether samples originate from the same distribution. If the test is statistically significant (*P* value less than 0.05), it means that at least one of the samples is different from the other samples. (XLSX 122 kb)

Additional file 3: ROC analysis discriminating OSCC vs normal healthy donors using easyROC as a webtool, showing the three best performing CpGs from each gene of 18 evaluated. Comparing OSCC vs normal healthy donors in 355 CpGs, the following epigenetically altered genes revealed high discrimination power: *ZAP70*, *ITGA4*, *KIF1A*, *PARP15*, *EPHX3*, *NTM*, *LRRTM1*, *FLI1*, *MIR193*, *LINC00599*, *PAX1*, and *MIR137HG* showing hypermethylation and *MIR296*, *TERT*, and *GP1BB* showing hypomethylation*.* (PDF 255 kb)

Additional file 4: Heatmap from 325 CpG methylation data points (rows) and 130 samples (column). Annotation labels refer to histology and smoking status. Rows are centered; unit variance scaling is applied to rows. Both rows and columns are clustered using correlation distance and average linkage. Smoking status and histology are highlighted in color. Three clusters are marked: left cluster: 55 normal donors, 21 contralateral mucosa, one OSCC, and the OSCC with sarcomatoid features; center cluster: three HGSIL, 11 OSCC, five contralateral mucosa, and one normal donor; right cluster: 16 OSCC, three HGSIL, four contralateral mucosa, and nine normal donors. (PDF 376 kb)

Additional file 5: *LINE1* mean methylation levels among OSCC, HGSIL, normal healthy donors, and contralateral normal mucosa. Asterisks indicate a statistical significance as calculated by the Kruskal-Wallis test. (PDF 22 kb)

Additional file 6: PCA for validation dataset: Unit variance scaling is applied to rows; SVD with imputation is used to calculate principal components. *X* and *Y* axes show principal component 1 and principal component 2 that explain 53.9 and 9.8% of the total variance, respectively. Prediction ellipses are such that with probability 0.95, a new observation from the same group will fall inside the ellipse. *N* = 40 data points. (PDF 29 kb)

Additional file 7: Heatmap for validation dataset. Rows are centered; unit variance scaling is applied to rows. Imputation is used for missing value estimation. Both rows and columns are clustered using correlation distance and average linkage; 54 rows, 40 columns. (PDF 45 kb)

## Abbreviations

HGSIL: High-grade squamous intraepithelial lesion
NGS: Next-generation sequencing
OLP: Oral lichen planus
OPML: Oral premalignant lesion
OSCC: Oral squamous cell carcinoma
ROC: Receiver operating characteristic

## References

[1] Warnakulasuriya S (2009). Global epidemiology of oral and oropharyngeal cancer. Oral Oncol.

[2] Arduino PG, Bagan J, El-Naggar AK, Carrozzo M (2013). Urban legends series: oral leukoplakia. Oral Dis.

[3] Scheifele C, Reichart PA (2003). Is there a natural limit of the transformation rate of oral leukoplakia?. Oral Oncol.

[4] Braakhuis BJ, Tabor MP, Leemans CR, van der Waal I, Snow GB, Brakenhoff RH (2002). Second primary tumors and field cancerization in oral and oropharyngeal cancer: molecular techniques provide new insights and definitions. Head Neck.

[5] Demokan S, Chang X, Chuang A, Mydlarz WK, Kaur J, Huang P, Khan Z, Khan T, Ostrow KL, Brait M (2010). KIF1A and EDNRB are differentially methylated in primary HNSCC and salivary rinses. Int J Cancer.

[6] Langevin SM, Stone RA, Bunker CH, Grandis JR, Sobol RW, Taioli E (2010). MicroRNA-137 promoter methylation in oral rinses from patients with squamous cell carcinoma of the head and neck is associated with gender and body mass index. Carcinogenesis.

[7] Pattani KM, Zhang Z, Demokan S, Glazer C, Loyo M, Goodman S, Sidransky D, Bermudez F, Jean-Charles G, McCaffrey T (2010). Endothelin receptor type B gene promoter hypermethylation in salivary rinses is independently associated with risk of oral cavity cancer and premalignancy. Cancer Prev Res (Phila).

[8] Schussel J, Zhou XC, Zhang Z, Pattani K, Bermudez F, Jean-Charles G, McCaffrey T, Padhya T, Phelan J, Spivakovsky S (2013). EDNRB and DCC salivary rinse hypermethylation has a similar performance as expert clinical examination in discrimination of oral cancer/dysplasia versus benign lesions. Clin Cancer Res.

[9] Nagata S, Hamada T, Yamada N, Yokoyama S, Kitamoto S, Kanmura Y, Nomura M, Kamikawa Y, Yonezawa S, Sugihara K (2012). Aberrant DNA methylation of tumor-related genes in oral rinse: a noninvasive method for detection of oral squamous cell carcinoma. Cancer.

[10] Sailer V, Holmes EE, Gevensleben H, Goltz D, Droge F, de Vos L, Franzen A, Schrock F, Bootz F, Kristiansen G (2016). PITX2 and PANCR DNA methylation predicts overall survival in patients with head and neck squamous cell carcinoma. Oncotarget.

[11] Herman JG, Baylin SB (2003). Gene silencing in cancer in association with promoter hypermethylation. N Engl J Med.

[12] Mishra R (2012). Biomarkers of oral premalignant epithelial lesions for clinical application. Oral Oncol.

[13] Gale N, Blagus R, El-Mofty SK, Helliwell T, Prasad ML, Sandison A, Volavsek M, Wenig BM, Zidar N, Cardesa A (2014). Evaluation of a new grading system for laryngeal squamous intraepithelial lesions—a proposed unified classification. Histopathology.

[14] The International Agency for Research on Cancer LB, J.W. Eveson, P. Reichart, D. Sidransky. World Health Organization classification of tumours: pathology and genetics of head and neck tumours. 1st edition. Lyon: IA RC Press; 2017.

[15] Morandi L, Gissi D, Tarsitano A, Asioli S, Monti V, Del Corso G, Marchetti C, Montebugnoli L, Foschini MP (2015). DNA methylation analysis by bisulfite next-generation sequencing for early detection of oral squamous cell carcinoma and high-grade squamous intraepithelial lesion from oral brushing. J Craniomaxillofac Surg.

[16] Thompson L (2006). World Health Organization classification of tumours: pathology and genetics of head and neck tumours. Ear Nose Throat J.

[17] Gale N, Zidar N, Poljak M, Cardesa A (2014). Current views and perspectives on classification of squamous intraepithelial lesions of the head and neck. Head Neck Pathol.

[18] Roh JL, Westra WH, Califano JA, Sidransky D, Koch WM (2012). Tissue imprint for molecular mapping of deep surgical margins in patients with head and neck squamous cell carcinoma. Head Neck.

[19] Marsit CJ, Christensen BC, Houseman EA, Karagas MR, Wrensch MR, Yeh RF, Nelson HH, Wiemels JL, Zheng S, Posner MR (2009). Epigenetic profiling reveals etiologically distinct patterns of DNA methylation in head and neck squamous cell carcinoma. Carcinogenesis.

[20] Guerrero-Preston R, Michailidi C, Marchionni L, Pickering CR, Frederick MJ, Myers JN, Yegnasubramanian S, Hadar T, Noordhuis MG, Zizkova V (2014). Key tumor suppressor genes inactivated by “greater promoter” methylation and somatic mutations in head and neck cancer. Epigenetics.

[21] Kozaki K, Imoto I, Mogi S, Omura K, Inazawa J (2008). Exploration of tumor-suppressive microRNAs silenced by DNA hypermethylation in oral cancer. Cancer Res.

[22] Li YF, Hsiao YH, Lai YH, Chen YC, Chen YJ, Chou JL, Chan MW, Lin YH, Tsou YA, Tsai MH (2015). DNA methylation profiles and biomarkers of oral squamous cell carcinoma. Epigenetics.

[23] Pickering CR, Zhang J, Yoo SY, Bengtsson L, Moorthy S, Neskey DM, Zhao M, Ortega Alves MV, Chang K, Drummond J (2013). Integrative genomic characterization of oral squamous cell carcinoma identifies frequent somatic drivers. Cancer Discov.

[24] el-Naggar AK, Lai S, Tucker SA, Clayman GL, Goepfert H, Hong WK, Huff V (1999). Frequent loss of imprinting at the IGF2 and H19 genes in head and neck squamous carcinoma. Oncogene.

[25] Foy JP, Pickering CR, Papadimitrakopoulou VA, Jelinek J, Lin SH, William WN, Frederick MJ, Wang J, Lang W, Feng L (2015). New DNA methylation markers and global DNA hypomethylation are associated with oral cancer development. Cancer Prev Res (Phila).

[26] Yakushiji T, Uzawa K, Shibahara T, Noma H, Tanzawa H (2003). Over-expression of DNA methyltransferases and CDKN2A gene methylation status in squamous cell carcinoma of the oral cavity. Int J Oncol.

[27] Langevin SM, Eliot M, Butler RA, Cheong A, Zhang X, McClean MD, Koestler DC, Kelsey KT (2015). CpG island methylation profile in non-invasive oral rinse samples is predictive of oral and pharyngeal carcinoma. Clin Epigenetics.

[28] Li LC, Dahiya R (2002). MethPrimer: designing primers for methylation PCRs. Bioinformatics.

[29] Morandi L, Righi A, Maletta F, Rucci P, Pagni F, Gallo M, Rossi S, Caporali L, Sapino A, Lloyd RV (2017). Somatic mutation profiling of hobnail variant of papillary thyroid carcinoma. Endocr Relat Cancer.

[30] Afgan E, Baker D, van den Beek M, Blankenberg D, Bouvier D, Cech M, Chilton J, Clements D, Coraor N, Eberhard C (2016). The Galaxy platform for accessible, reproducible and collaborative biomedical analyses: 2016 update. Nucleic Acids Res.

[31] Hu K, Ting AH, Li J (2015). BSPAT: a fast online tool for DNA methylation co-occurrence pattern analysis based on high-throughput bisulfite sequencing data. BMC Bioinf.

[32] Lutsik P, Feuerbach L, Arand J, Lengauer T, Walter J, Bock C (2011). BiQ Analyzer HT: locus-specific analysis of DNA methylation by high-throughput bisulfite sequencing. Nucleic Acids Res.

[33] Kumaki Y, Oda M, Okano M (2008). QUMA: quantification tool for methylation analysis. Nucleic Acids Res.

[34] Rohde C, Zhang Y, Reinhardt R, Jeltsch A (2010). BISMA—fast and accurate bisulfite sequencing data analysis of individual clones from unique and repetitive sequences. BMC Bioinformatics.

[35] Mallona I, Diez-Villanueva A, Peinado MA (2014). Methylation plotter: a web tool for dynamic visualization of DNA methylation data. Source Code Biol Med.

[36] Metsalu T, Vilo J (2015). ClustVis: a web tool for visualizing clustering of multivariate data using principal component analysis and heatmap. Nucleic Acids Res.

[37] Akers SN, Moysich K, Zhang W, Collamat Lai G, Miller A, Lele S, Odunsi K, Karpf AR (2014). LINE1 and Alu repetitive element DNA methylation in tumors and white blood cells from epithelial ovarian cancer patients. Gynecol Oncol.

[38] King OH (1971). Cytology—its value in the diagnosis of oral cancer. Dent Clin N Am.

[39] Brocklehurst P, Kujan O, O’Malley LA, Ogden G, Shepherd S, Glenny AM (2013). Screening programmes for the early detection and prevention of oral cancer. Cochrane Database Syst Rev.

[40] Sandoval J, Esteller M (2012). Cancer epigenomics: beyond genomics. Curr Opin Genet Dev.

[41] van Vlodrop IJ, Niessen HE, Derks S, Baldewijns MM, van Criekinge W, Herman JG, van Engeland M (2011). Analysis of promoter CpG island hypermethylation in cancer: location, location, location!. Clin Cancer Res.

[42] Dang J, Bian YQ, Sun JY, Chen F, Dong GY, Liu Q, Wang XW, Kjems J, Gao S, Wang QT (2013). MicroRNA-137 promoter methylation in oral lichen planus and oral squamous cell carcinoma. J Oral Pathol Med.

[43] Fitzpatrick SG, Hirsch SA, Gordon SC (2014). The malignant transformation of oral lichen planus and oral lichenoid lesions: a systematic review. J Am Dent Assoc.

[44] BLUEPRINT Consortium, Bock C, Halbritter F, Carmona FJ, et al. Quantitative comparison of DNA methylation assays for biomarker development and clinical applications. Nat Biotechnol. 2016;34(7):726–737. 10.1038/nbt.3605 27347756

[45] Kerkel K, Spadola A, Yuan E, Kosek J, Jiang L, Hod E, Li K, Murty VV, Schupf N, Vilain E (2008). Genomic surveys by methylation-sensitive SNP analysis identify sequence-dependent allele-specific DNA methylation. Nat Genet.

[46] Ogino S, Kawasaki T, Nosho K, Ohnishi M, Suemoto Y, Kirkner GJ, Fuchs CS (2008). LINE-1 hypomethylation is inversely associated with microsatellite instability and CpG island methylator phenotype in colorectal cancer. Int J Cancer.

[47] Afgan E, Baker D, van den Beek M, Blankenberg D, Bouvier D, Cech M, Chilton J, Clements D, Coraor N, Eberhard C et al. The Galaxy platform for accessible, reproducible and collaborative biomedical analyses: 2016 update. Nucleic Acids Res. 2016;44(W1):W3–W10. doi:10.1093/nar/gkw343. 10.1093/nar/gkw343 PMC4987906 27137889

[48] Bock C, Reither S, Mikeska T, Paulsen M, Walter J, Lengauer T (2005). BiQ Analyzer: visualization and quality control for DNA methylation data from bisulfite sequencing. Bioinformatics.

[49] Zackay A, Steinhoff C (2010). MethVisual—visualization and exploratory statistical analysis of DNA methylation profiles from bisulfite sequencing. BMC Res Notes.

[50] Krueger F, Andrews SR (2011). Bismark: a flexible aligner and methylation caller for Bisulfite-Seq applications. Bioinformatics.

[51] Chen PY, Cokus SJ, Pellegrini M (2010). BS Seeker: precise mapping for bisulfite sequencing. BMC Bioinformatics.

[52] Huang Z, Zhu D, Wu L, He M, Zhou X, Zhang L, Zhang H, Wang W, Zhu J, Cheng W (2017). Six serum-based miRNAs as potential diagnostic biomarkers for gastric cancer. Cancer Epidemiol Biomark Prev.

[53] Nordentoft I, Birkenkamp-Demtroder K, Agerbaek M, Theodorescu D, Ostenfeld MS, Hartmann A, Borre M, Orntoft TF, Dyrskjot L (2012). miRNAs associated with chemo-sensitivity in cell lines and in advanced bladder cancer. BMC Med Genet.

[54] Luo W, Lin Y, Meng S, Guo Y, Zhang J, Zhang W (2016). miRNA-296-3p modulates chemosensitivity of lung cancer cells by targeting CX3CR1. Am J Transl Res.

[55] Nagarajan RP, Zhang B, Bell RJ, Johnson BE, Olshen AB, Sundaram V, Li D, Graham AE, Diaz A, Fouse SD (2014). Recurrent epimutations activate gene body promoters in primary glioblastoma. Genome Res.

[56] Jiang J, Zhao LJ, Zhao C, Zhang G, Zhao Y, Li JR, Li XP, Wei LH (2012). Hypomethylated CpG around the transcription start site enables TERT expression and HPV16 E6 regulates TERT methylation in cervical cancer cells. Gynecol Oncol.

[57] Savoia A, Pastore A, De Rocco D, Civaschi E, Di Stazio M, Bottega R, Melazzini F, Bozzi V, Pecci A, Magrin S (2011). Clinical and genetic aspects of Bernard-Soulier syndrome: searching for genotype/phenotype correlations. Haematologica.

[58] Claus R, Lucas DM, Ruppert AS, Williams KE, Weng D, Patterson K, Zucknick M, Oakes CC, Rassenti LZ, Greaves AW (2014). Validation of ZAP-70 methylation and its relative significance in predicting outcome in chronic lymphocytic leukemia. Blood.

[59] Okada Y, Yamazaki H, Sekine-Aizawa Y, Hirokawa N (1995). The neuron-specific kinesin superfamily protein KIF1A is a unique monomeric motor for anterograde axonal transport of synaptic vesicle precursors. Cell.

[60] Sheffield NC, Pierron G, Klughammer J, Datlinger P, Schonegger A, Schuster M, Hadler J, Surdez D, Guillemot D, Lapouble E et al. DNA methylation heterogeneity defines a disease spectrum in Ewing sarcoma. Nat Med. 2017;23(3):386–395. doi:10.1038/nm.4273. 10.1038/nm.4273 PMC5951283 28134926

[61] Vymetalkova V, Vodicka P, Pardini B, Rosa F, Levy M, Schneiderova M, Liska V, Vodickova L, Nilsson TK, Farkas SA (2016). Epigenome-wide analysis of DNA methylation reveals a rectal cancer-specific epigenomic signature. Epigenomics.

[62] Sepulveda JL, Gutierrez-Pajares JL, Luna A, Yao Y, Tobias JW, Thomas S, Woo Y, Giorgi F, Komissarova EV, Califano A (2016). High-definition CpG methylation of novel genes in gastric carcinogenesis identified by next-generation sequencing. Mod Pathol.

[63] Pelch KE, Tokar EJ, Merrick BA, Waalkes MP (2015). Differential DNA methylation profile of key genes in malignant prostate epithelial cells transformed by inorganic arsenic or cadmium. Toxicol Appl Pharmacol.

[64] Bell A, Bell D, Weber RS, El-Naggar AK (2011). CpG island methylation profiling in human salivary gland adenoid cystic carcinoma. Cancer.

[65] Lam K, Pan K, Linnekamp JF, Medema JP, Kandimalla R (2016). DNA methylation based biomarkers in colorectal cancer: a systematic review. Biochim Biophys Acta.

[66] Jian B, Li Z, Xiao D, He G, Bai L, Yang Q (2016). Downregulation of microRNA-193-3p inhibits tumor proliferation migration and chemoresistance in human gastric cancer by regulating PTEN gene. Tumour Biol.

[67] Slaughter DP, Southwick HW, Smejkal W (1953). Field cancerization in oral stratified squamous epithelium; clinical implications of multicentric origin. Cancer.

[68] Braakhuis BJ, Tabor MP, Kummer JA, Leemans CR, Brakenhoff RH (2003). A genetic explanation of Slaughter’s concept of field cancerization: evidence and clinical implications. Cancer Res.

[69] Griffioen GH, Louie AV, de Bree R, Smit EF, Paul MA, Slotman BJ, Leemans CR, Senan S (2015). Second primary lung cancers following a diagnosis of primary head and neck cancer. Lung Cancer.

