# Supplementary figures and images for: CpG location and methylation level are crucial factors for the early detection of oral squamous cell carcinoma in brushing samples using bisulfite sequencing of a 13-gene panel
Source: Clin Epigenetics. 2017 Aug 15;9:85. doi: 10.1186/s13148-017-0386-7 (PMC5558660; doi:10.1186/s13148-017-0386-7)

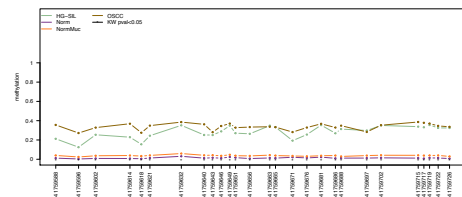

**KIF1A**

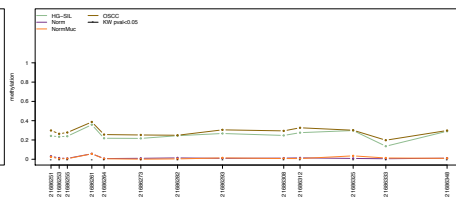

**PAX1**

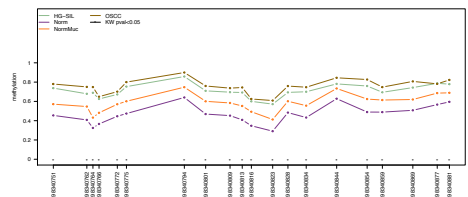

**ZAP70**

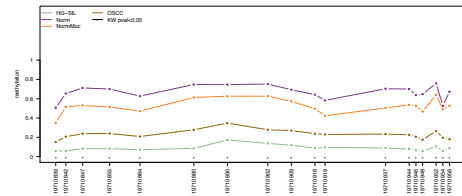

**GP1BB**

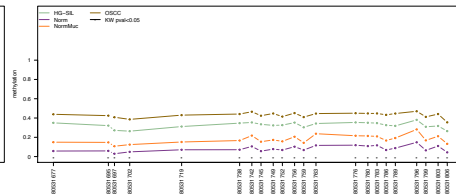

**LRRTM1**

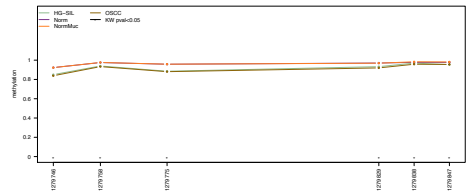

**TERT**

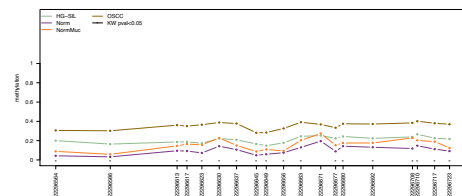

**PARP15**

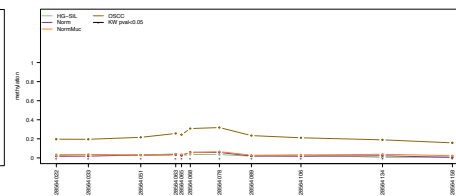

**FLI1**

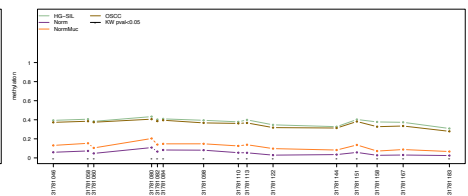

**NTM**

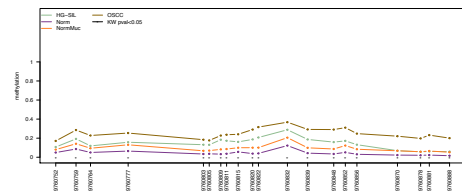

**LINC0059**

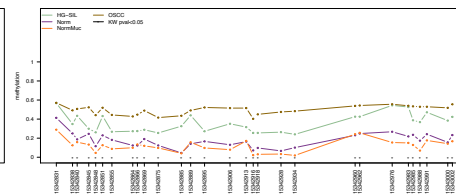

**EPHX3**

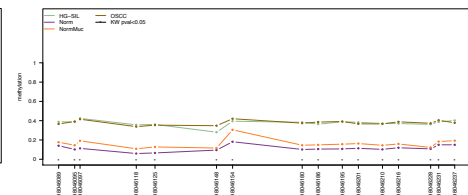

**MIR137**

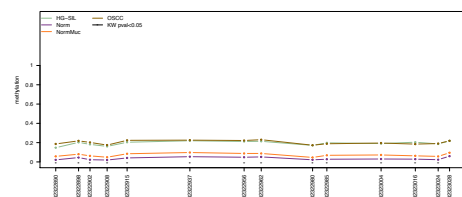

**ITGA4**

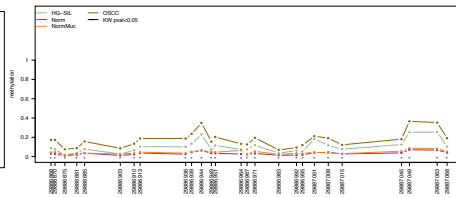

**MIR193**

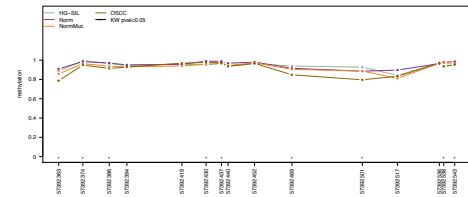

**MIR296**

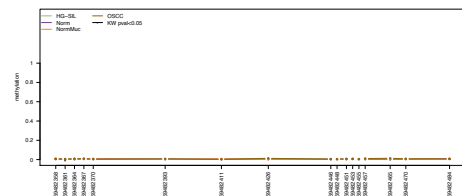

**TERC**

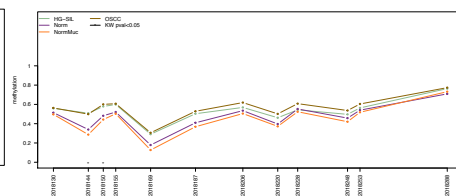

**H19**

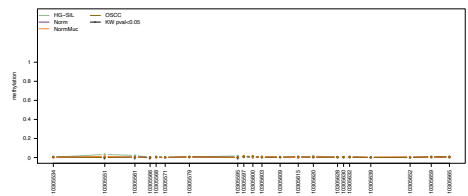

**DNMT1**

Supplement: Supplementary file 1 — Methylation profile plot from 18 genes evaluated. For each group of samples, each line represents the methylation mean for each position. Asterisks indicate a statistical significance as calculated by the Kruskal-Wallis test. ZAP70, GP1BB, H19, EPHX3, and MIR193 revealed a fluctuating behavior among the various CpGs evaluated. The gap between normal and OSCC remained mostly the same (Kruskal-Wallis P values were < 0.05), but the absolute values changed conspicuously among different positions investigated. (PDF 415 kb) [file 13148_2017_386_MOESM1_ESM.pdf]

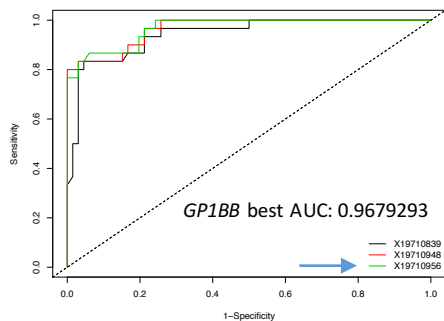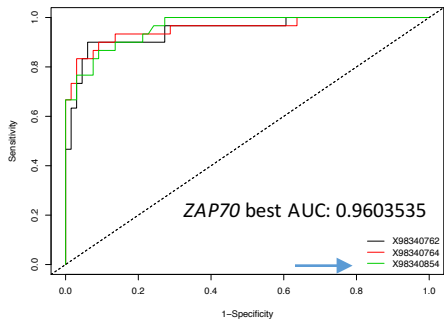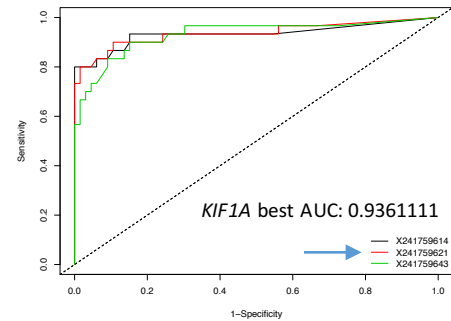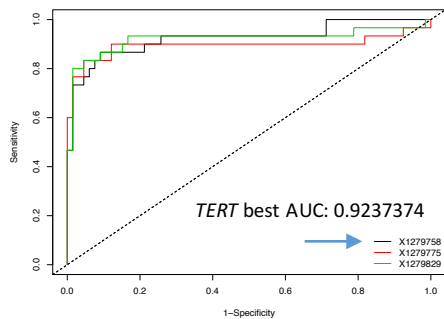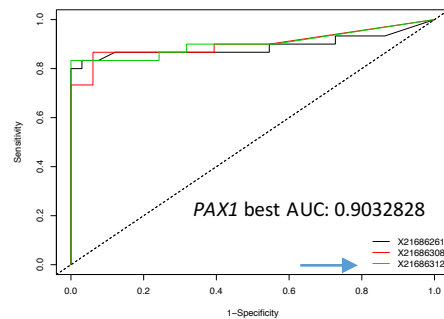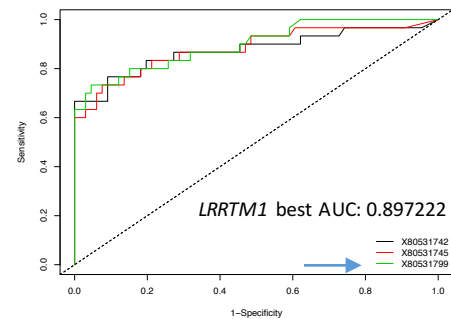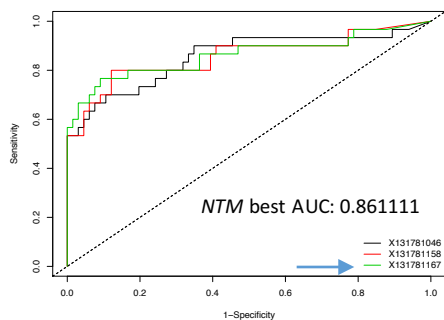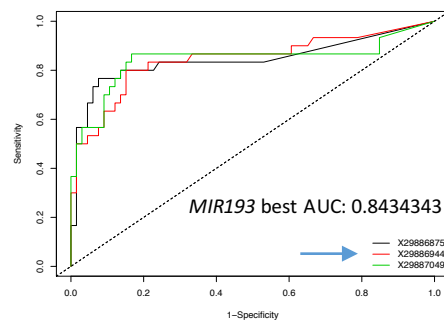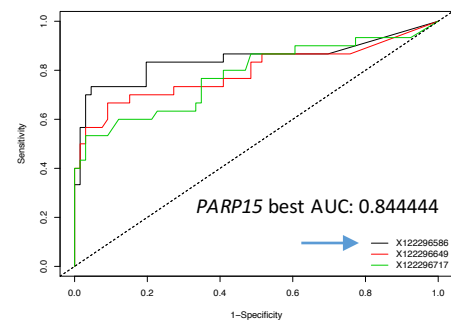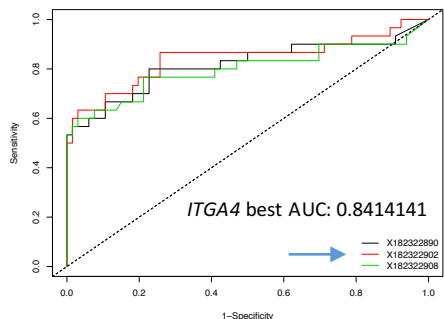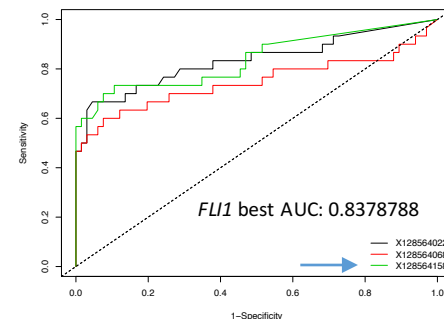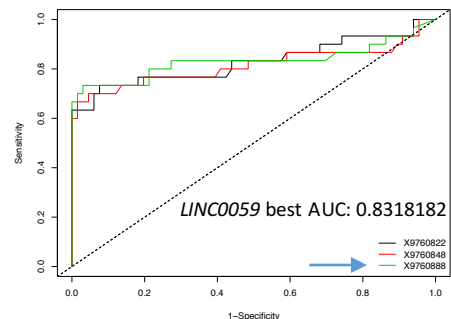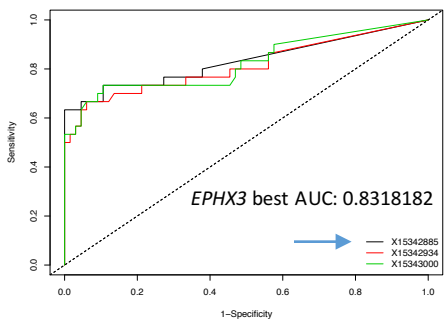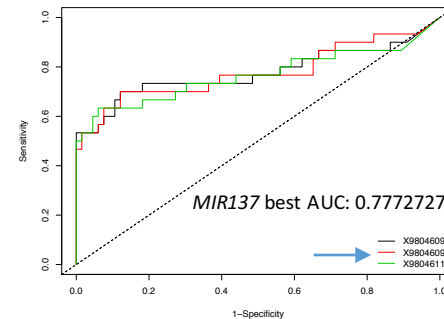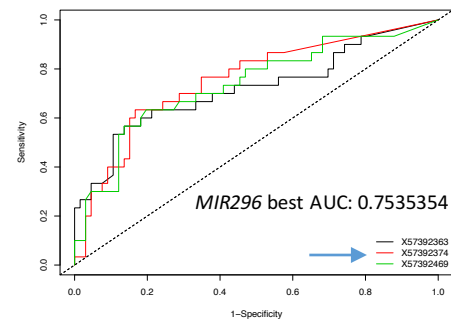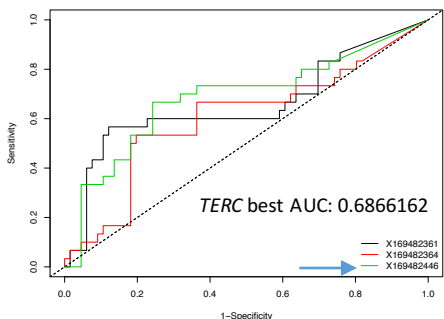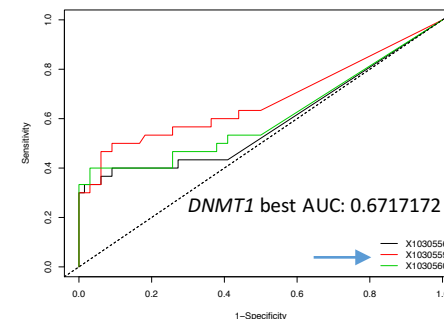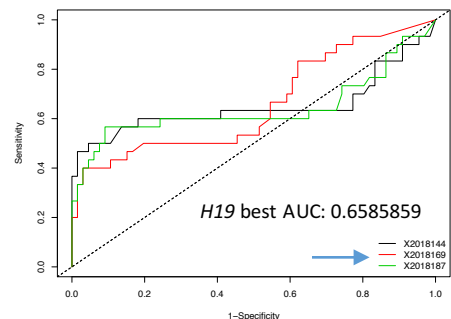

Supplement: Supplementary file 3 — ROC analysis discriminating OSCC vs normal healthy donors using easyROC as a webtool, showing the three best performing CpGs from each gene of 18 evaluated. Comparing OSCC vs normal healthy donors in 355 CpGs, the following epigenetically altered genes revealed high discrimination power: ZAP70, ITGA4, KIF1A, PARP15, EPHX3, NTM, LRRTM1, FLI1, MIR193, LINC00599, PAX1, and MIR137HG showing hypermethylation and MIR296, TERT, and GP1BB showing hypomethylation. (PDF 255 kb) [file 13148_2017_386_MOESM3_ESM.pdf]

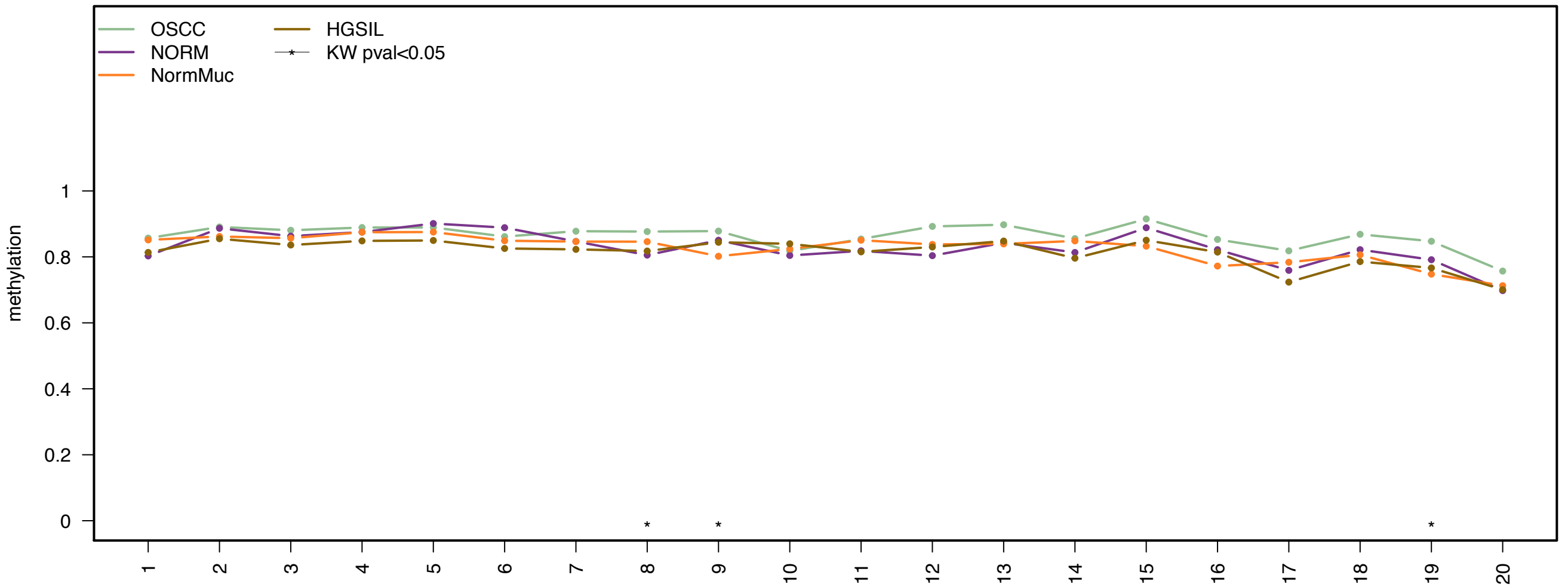

Supplement: Supplementary file 5 — LINE1 mean methylation levels among OSCC, HGSIL, normal healthy donors, and contralateral normal mucosa. Asterisks indicate a statistical significance as calculated by the Kruskal-Wallis test. (PDF 22 kb) [file 13148_2017_386_MOESM5_ESM.pdf]

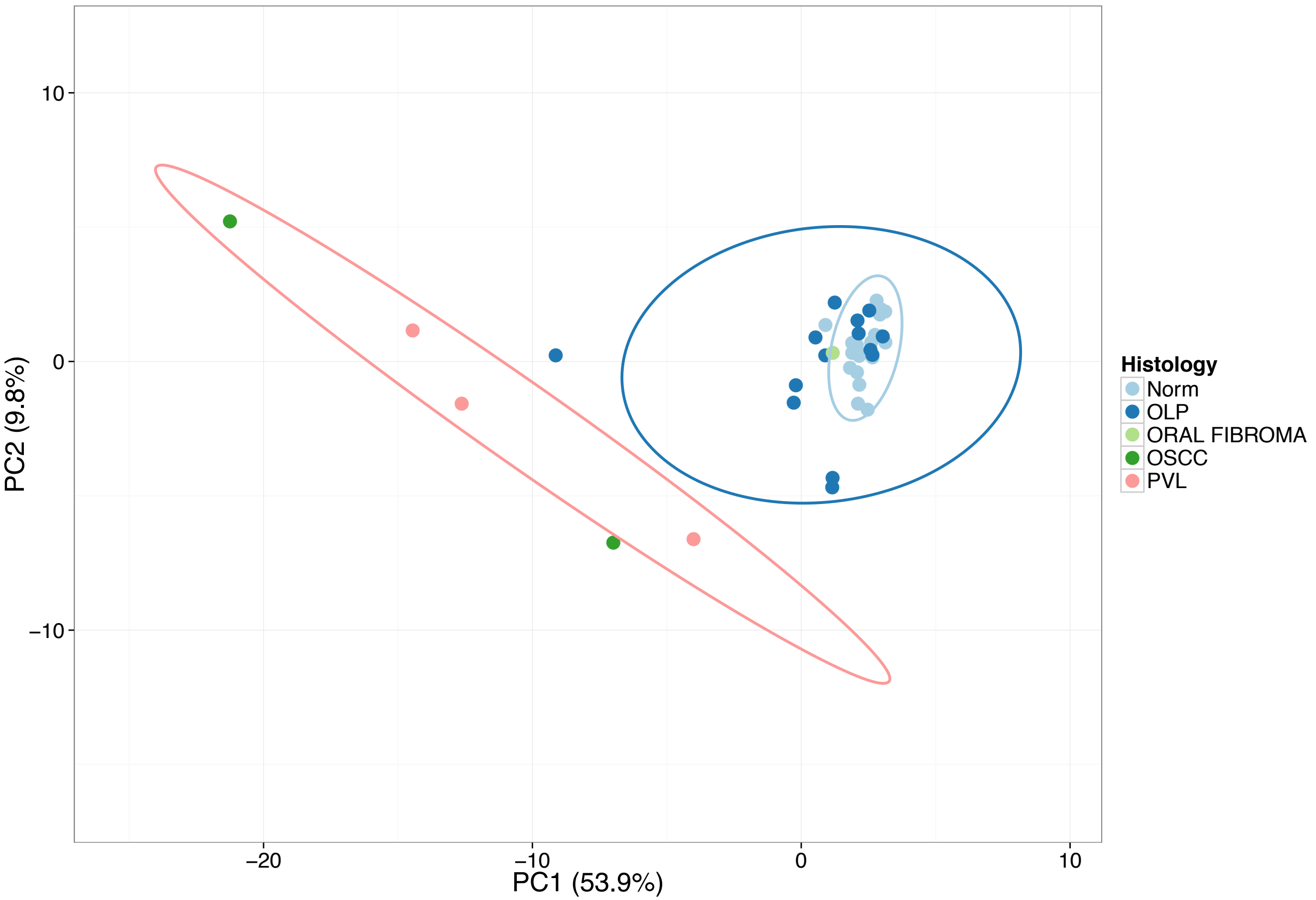

Supplement: Supplementary file 6 — PCA for validation dataset: Unit variance scaling is applied to rows; SVD with imputation is used to calculate principal components. X and Y axes show principal component 1 and principal component 2 that explain 53.9 and 9.8% of the total variance, respectively. Prediction ellipses are such that with probability 0.95, a new observation from the same group will fall inside the ellipse. N = 40 data points. (PDF 29 kb) [file 13148_2017_386_MOESM6_ESM.pdf]

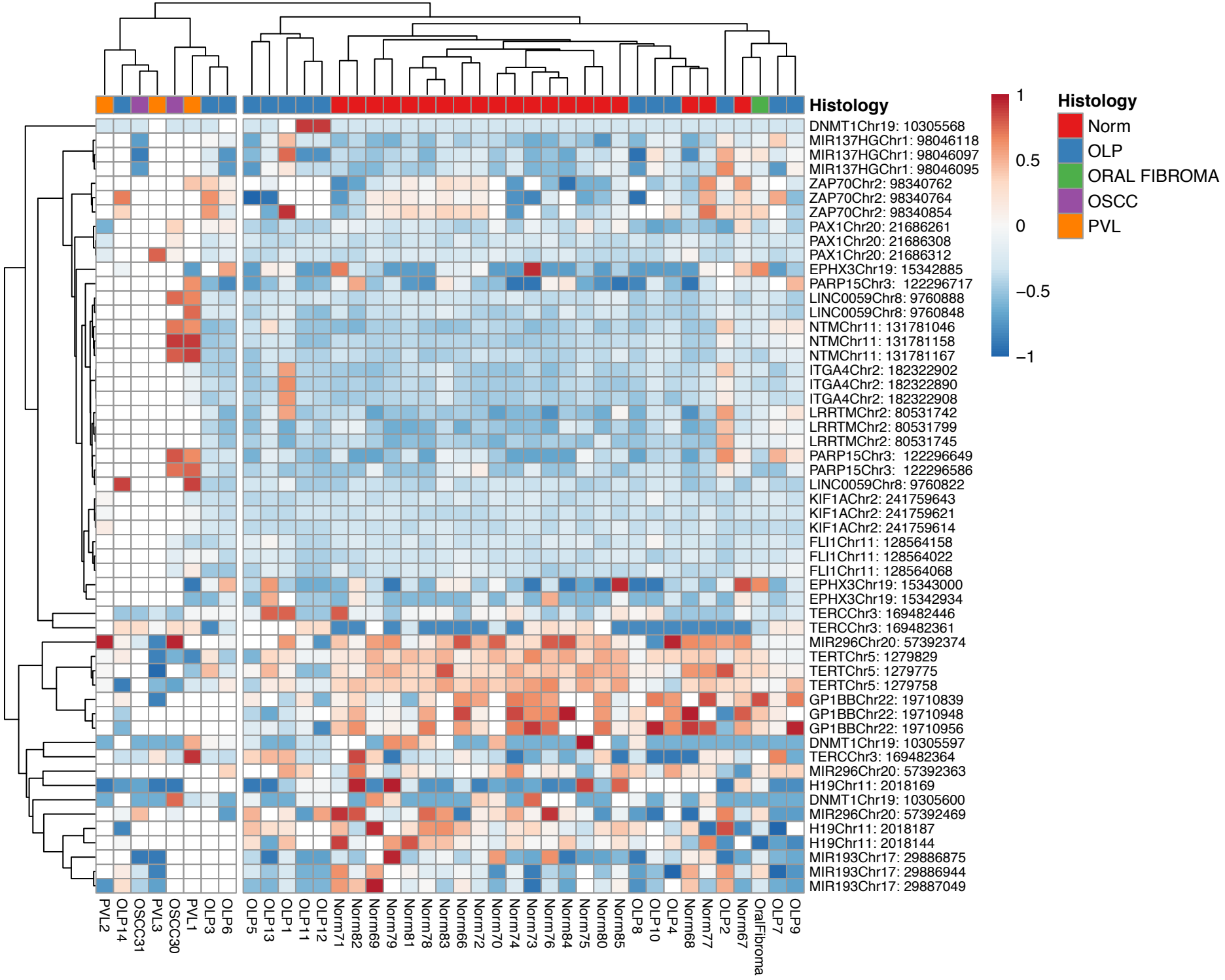

Supplement: Supplementary file 7 — Heatmap for validation dataset. Rows are centered; unit variance scaling is applied to rows. Imputation is used for missing value estimation. Both rows and columns are clustered using correlation distance and average linkage; 54 rows, 40 columns. (PDF 45 kb) [file 13148_2017_386_MOESM7_ESM.pdf]
